# Sequence-dependent histone variant positioning signatures

**DOI:** 10.1186/1471-2164-11-S4-S3

**Published:** 2010-12-02

**Authors:** Ngoc Tu Le, Tu Bao Ho, Bich Hai Ho

**Affiliations:** 1 School of Knowledge Science, Japan Advanced Institute of Science and Technology, 1-1 Asahidai, Nomi, Ishikawa 923-1292, Japan; 2 Hanoi National University of Education, 136 Xuan Thuy, Cau Giay, Hanoi, Vietnam; 3 Vietnamese Academy of Science and Technology, 18 Hoang Quoc Viet, Cau Giay, Hanoi, Vietnam

## Abstract

**Background:**

Nucleosome, the fundamental unit of chromatin, is formed by wrapping nearly 147bp of DNA around an octamer of histone proteins. This histone core has many variants that are different from each other by their biochemical compositions as well as biological functions. Although the deposition of histone variants onto chromatin has been implicated in many important biological processes, such as transcription and replication, the mechanisms of how they are deposited on target sites are still obscure.

**Results:**

By analyzing genomic sequences of nucleosomes bearing different histone variants from human, including H2A.Z, H3.3 and both (H3.3/H2A.Z, so-called double variant histones), we found that genomic sequence contributes in part to determining target sites for different histone variants. Moreover, dinucleotides CA/TG are remarkably important in distinguishing target sites of H2A.Z-only nucleosomes with those of H3.3-containing (both H3.3-only and double variant) nucleosomes.

**Conclusions:**

There exists a DNA-related mechanism regulating the deposition of different histone variants onto chromatin and biological outcomes thereof. This provides additional insights into epigenetic regulatory mechanisms of many important cellular processes.

## Background

Eukaryotic genomes are packaged into chromatin, a highly condensed structure like a bead-on-string fiber with fundamental repeating units, the nucleosomes. Each nucleosome is formed by wrapping 147bp of DNA around a histone core, an octamer of proteins that contains a central (*H*3 – *H*4)_2_ tetramer flanked on both side by two *H*2*A* – *H*2*B* dimers [1]. Biological evidences have increasingly shown that, far beyond simple DNA compaction chromatin imposes ubiquitous and profound effects on many important DNA-based processes, such as transcription, DNA replication and repair [2-5]. During those processes, the structure of chromatin must be dynamically and reversibly altered to enable or repress the access of cellular machineries to underlying genomic sequences.

To alter histone-DNA and histone-histone interactions the cell has developed diverse and complicated pathways, such as post-translational modifications (PTMs) of histone proteins, ATP-dependent chromatin remodeling or replacement of canonical histones by nonallelic histone variants [6]. For example, changes in the net charge of nucleosomes by lysine acetylation would result in increasing nucleosome mobility, making acetylated nucleosomes easier to displace from their translational positions [7-9]; in yeast, nucleosomes can be moved from their default positions to energetically unfavorable positions by ISW2 ATP-dependent chromatin remodeling complex or even evicted from chromatin by orchestrated action of RSC chromatin remodeling complex and nucleosome-assembly protein 1 (Nap1) histone chaperone [10,11]; in human, nucleosomes bearing both H2A.Z and H3.3 histone variants are observed to be prone to eviction [12]. Despite their important roles in regulating chromatin structure and consequently chromatin-based processes, not until recently have histone variants received considerable attention. Among the most extensively studied histone variants are H3.3 and H2A.Z, the isoforms of histones H3 and H2A, respectively. H3.3 has been implicated in many biological pathways in which it may function as neutral replacement histone at chromatin regions where histones have been displaced upon transcriptional activities [13], or it may help to transmit epigenetic memory of active gene states [14]. Recent studies have also revealed unrecognized roles of this variant in chromatin remodeling mechanisms during sexual reproduction [15] and in forming open chromatin regions by inhibiting the binding of linker histone H1 [16]. While studies on H3.3 highly agree about its functions, controversial characteristics have been observed for H2A.Z [17,18]. In some cases, H2A.Z is shown to increase the stability of nucleosomes bearing it [19] while in others it appears that H2A.Z is easier to displace from chromatin than is H2A [20,21]; H2A.Z has also been claimed to involve in gene inactivation [22] and activation (or both) [23]. Nevertheless, most of previous works came into the same conclusion that H2A.Z and H3.3 are non-randomly distributed along the genome and those distributions may reflect their biological functions [24]. So understanding how H3.3 and H2A.Z are distributed over the genome and its biological implication gives us deeper insights into epigenetic mechanisms of many important processes.

Previous works have shown that, histone variants differ from their canonical counterparts not only by their biochemical compositions, ranging from a few amino-acid positions to large protein domains, but also by their incorporations into chromatin. While canonical histones are expressed and incorporated into chromatin during DNA replication in S phase, most histone variants are synthesized throughout the cell cycle and available to nucleosome assembly pathways that occur in a Replication-Independent (RI) manner [13,25]. For example, it has been known in [26-28] that Swi2/Snf2-related ATP-dependent chromatin remodeling complex SWR1, histone chaperones Nap1 and Chz1 involve in the assembly of Htz1 (H2A.Z in yeast), while the deposition of H3.3 involves with the activity of histone chaperone HIRA [29]. It still remains elusive, however, that how histone variants H2A.Z and H3.3 are targeted to the deposited sites [24,25,30].

The advancement of high-throughput profiling technologies such as ChIP-Chip and ChIP-Seq makes it possbile to map genome-wide distributions of nucleosomes bearing different histone variants [21,31]. This offers an unprecedented opportunity to investigate the effects of histone variant distribution on cellular processes (e.g. transcription) as well as its regulatory factors. More recently, Jin et al. [32] have investigated genome-wide distributions of nucleosomes containing different histone variants, including H2A.Z, H3.3 and both (H3.3/H2A.Z, so-called double variant histones) in human genome. The work has shown that nucleosomes bearing double variant histones mainly account for the enriched patterns of histone variants observed at promoters and other important regulatory regions of active genes. Using the data available from Jin et al. [32], we investigated whether there exist genomic features that may help to distinguish target sites of nucleosomes bearing different histone variants in the vicinity of promoter regions. We found that genomic sequence contributes partially to determining the target sites of these variants. Moreover, dinucleotides CA/TG are remarkably important in distinguishing target sites of H2A.Z-only nucleosomes with those of H3.3-containing (both H3.3-only and double variant) nucleosomes. These results give additional insights into epigenetic regulatory mechanisms of many important cellular processes.

## Results and discussion

### Genomic sequence partially distinguishes target sites of double variant nucleosomes with those of nucleosomes bearing only H3.3 or H2A.Z

High-resolution studies on crystal structure of the nucleosome core particle have revealed that the DNA is wrapped around the histone octamer in a flat, left-handed superhelix [1]. Based on this finding, the notion of sequence-dependent affinity of the histone core is defined as the energetic cost to bend the DNA to accommodate the superhelical path. The observation that nucleosomes show higher affinity for particular DNA sequences [33] has fostered many efforts to find genomic signatures related to genome-wide nucleosome positioning [34-37]. Although the role of genomic sequence on nucleosome distribution is still a controversial topic, it has been confirmed, both *in vivo* and *in vitro,* that DNA sequence imparts nucleosome distribution and there actually exists genomic code for nucleosome positioning [38-40]. Regarding important functions of H2A.Z, many efforts have been spent on identifying genomic signatures that may affect its genome-wide distribution. Tolstorukov et al. [41] compared genomic sequences of H2A.Z-containing nucleosomes from yeast and human and concluded that human nucleosomal sequences do not show the pattern of 10-bp periodicity as observed in yeast. Despite trying a handful of approaches, Gervais et al. [42] could not find any specific DNA motif that can help to distinguish H2A.Z-containing nucleosomal sequences from those of nucleosomes containing canonical histones. Other computational approaches also failed to distinguish H2A.Z-containing nucleosomal sequences from linker sequences [37]. Recently, the work of Jin et al. [32] gives the evidence that most of H2A.Z-containing nucleosomes observed at important genomic regions, such as promoters, CTCF binding sites, etc. are actually double variant nucleosomes. Taken together, we speculate that at important genomic regions (e.g. promoters) although histone variant-containing nucleosomes do not occupy thermodynamically favorite locations, DNA sequence may help to distinguish target sites of double variant nucleosomes from those of nucleosomes bearing only H2A.Z or H3.3.

To verify this hypothesis, we applied the computational procedure proposed by Peckham et al. [36] to the problem of discriminating double variant nucleosomal sequences from H2A.Z-only or H3.3-only nucleosomal sequences. This method has shown competitive performance for the task of discriminating “nucleosome forming” sequences from “nucleosome inhibiting” sequences and was applied sucessfully on human data [43]. The SVM classifiers in our work were built using *Radial Basis Function (RBF)* kernel (see *Methods)* instead of linear (dot product) kernel as used in original work. Sequence datasets, called *double-h2az* and *double-h33* for training SVM classifiers were created as described in *Methods.* Each sequence in the training sets was represented as a 2, 772-entry vector, in which each entry is a normalized count of the occurences of a particular *k-mer* or its reverse complement, with *k=1* up to 6. These vectors were used to train SVM classifiers.

To evaluate the performances of the resultant classifiers we used 10-fold cross-validation procedure. According to this procedure, each dataset was divided randomly into 10 subsets. The classifiers were trained on 9 subsets and tested on the remaining one. This training-testing procedure was repeated 10 times using a different hold-out set at each time. To measure the performances of the classifiers, we utilized the *receive-operator-characteristic* (ROC) curve. The quality of the classifier can be evaluated by calculating the *area-under-the-curve* (AUC) (the “ROC score”), in which a random classifier achieves the ROC score of 0.5 and a perfect classifier achieves the ROC score of 1.0. The average ROC scores from 10-fold cross validation on two sequence datasets, *double-h2az* and *double-h33,* were 0.62 (*SD* ≈ 0.02) and 0.63 (*SD* ≈ 0.03), respectively. These are significantly higher than the performance of the random classifier (*p* = 0.3 × 10^–5^ and *p* = 0.15 × 10^–4^ (*t*-test), correspondingly). This result shows that, genomic sequence contributes in part to targeting double variant nucleosomes to sites different from those of H2A.Z-only and H3.3-only nucleosomes.

### Characteristics of sequences wrapping diffenrent histone variants

The result above suggests us to search for sequence features characterizing target sites of different histone variants. Basically, there are two kinds of such features [44]: one is compositional discriminative motif, which may help to distinguish double variant nucleosomal sequences from H2A.Z-only and H3.3-only ones; and the other is periodic pattern, which may appear in the set of nucleosomal sequences.

There are several approaches to find compositional discriminative motifs. For example, Peckham et al. [36] used a simple word counting method to compute the frequencies of different DNA motifs and evaluated discriminative power of each motif in separating “nucleosome forming” sequences from “nucleosome inhibiting” ones based on its ROC score. Gupta et al. [43] computed the percentages of dinucleotides from “nucleosome forming” and “nucleosome inhibiting” sequence sets and found an overrepresentation of several dinucleotides in “nucleosome inhibitory” and “nucleosome favorable” sequences, such as AC/GT and CC/GG, correspondingly. In our work, we used feature selection with Fisher criterion, a simple yet effective method (see *Methods),* for the task. Two sets of normalized count vectors corresponding to *double-h2az* and *double-h33* datasets were used to evaluate the discriminative power of different DNA motifs. Analyzing the numbers of occurences of 20 strongest discriminative motifs in *double-h2a.z* (Table 1, ranked by F-score), we found that H2A.Z-only nucleosomal sequences are richer in AT-related motifs (e.g. AAT/ATT, TA, AA/TT, AAA/TTT), which are known to be nucleosome inhibitory signals, while double variant nucleosomal sequences are richer in such motifs as CAG/CTG, CA/TG, C/G, which are known to be nucleosome favoring signals [36]. For 20 strongest discriminative motifs in *double-h3.3* (Table 2, ranked by F-score), H3.3-only nucleosomal sequences are richer in highly flexible, nucleosome favoring motifs, such as CA/TG, ACA/TGT, CACA/TGTG, while double variant nuclesomal sequences are richer in nucleosome favoring but less flexible motifs, such as CC/GG.

**Table 1:** Strongest discriminative motifs corresponding to double-h2az dataset ranked by F-scores

Order	Motifs	F-score	Richer in H3.3/H2A.Z (+) or H2A.Z-only (-) nucleosomal sequences
1	CAG	0.0961057	+
2	AAT	0.079221	-
3	TA	0.0781893	-
4	CA	0.0715567	+
5	CAGG	0.0713273	+
6	AA	0.0709853	-
7	TAA	0.0662379	-
8	AATA	0.0655541	-
9	C	0.0649125	+
10	AAAT	0.0630749	-
11	ATA	0.0595751	-
12	ATAA	0.0559362	-
13	AAA	0.0554242	-
14	AGG	0.0551005	+
15	AG	0.0541048	+
16	AAAAT	0.0516458	-
17	TAAA	0.051629	-
18	CC	0.0491037	+
19	AAATA	0.0459333	-
20	AT	0.044603	-

**Table 2:** Strongest discriminative motifs corresponding to double-h33 dataset ranked by F-scores

Order	Motifs	F-score	Richer in H3.3/H2A.Z (+) or H3.3-only (-) nucleosomal sequences
1	GGA	0.08384	+
2	CA	0.0709705	-
3	GGAA	0.0655294	+
4	ACTCCC	0.0649791	+
5	ACA	0.0625949	-
6	AC	0.0595409	-
7	AGGA	0.0571402	+
8	GCTCC	0.0509788	+
9	ACAT	0.0499685	-
10	ATG	0.0499393	-
11	CTCCC	0.0469626	+
12	CC	0.0445543	+
13	CAC	0.0441645	-
14	CTCC	0.0428833	+
15	GGAAA	0.0427223	+
16	ACACA	0.0406986	-
17	GGGA	0.039697	+
18	CCCAGG	0.0386637	+
19	TGGAAA	0.0379053	+
20	CACA	0.0375203	-

**Figure 1:**
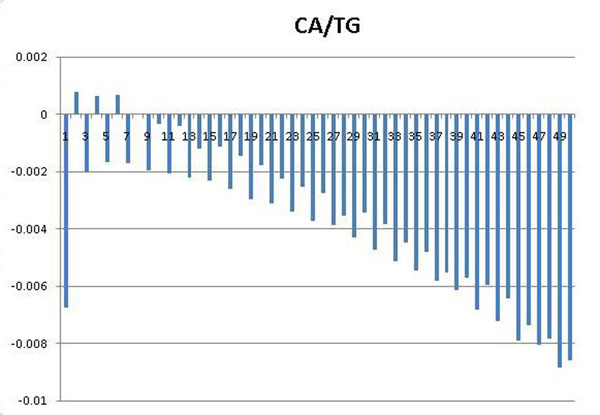
**Periodograms of dinucleotides CA/TG with *x*-axis representing distance *k*, *y*-axis representing average covariance value *C_XX_*(*k*)**. CA/TG periodicities corresponding to the set of double variant nucleosomal sequences

**Figure 2:**
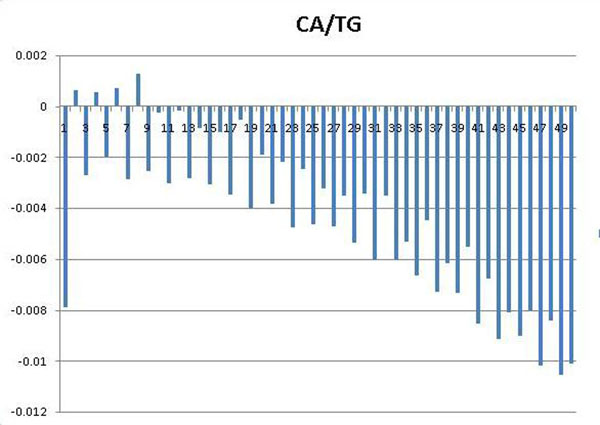
**Periodograms of dinucleotides CA/TG with *x*-axis representing distance *k*, *y*-axis representing average covariance value *C_XX_*(*k*).** CA/TG periodicities corresponding to the set of H3.3-only nucleosomal sequences

**Figure 3:**
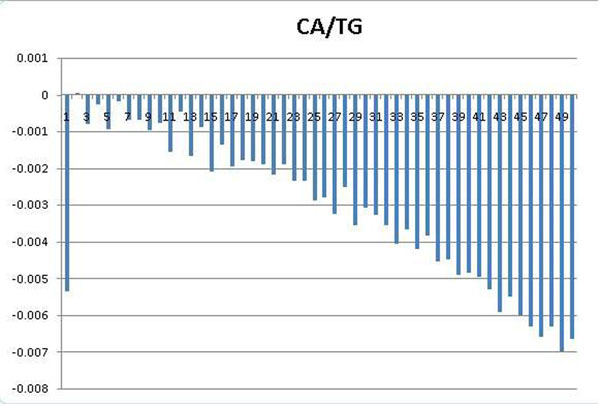
**Periodograms of dinucleotides CA/TG with *x*-axis representing distance *k*, *y*-axis representing average covariance value *C_XX_*(*k*).** CA/TG show no specific periodicity among the set of H2A.Z-only nucleosomal sequences

The periodicity has been known as one of the fundamental features that may appear in a set of DNA sequences. For example, 3bp sequence period is known to characterize coding sequences [45], while ~10bp sequence period is known to affect the curvature, bendability [46] and establish rotational setting on the histone surface [31] of the DNA sequences. To identify periodic patterns that may appear in nuclesomal sequences wrapping different histone variants, we employed autocorrelation analysis, which was sucessfully applied to detect hidden sequence periodicities in sets of DNA sequences [47,48](see *Methods).* Investigating the periodograms of DNA motifs which show strongest discriminative power, we found clear periods of 2, 4, 6 of dinucleotides CA/TG in the set of 2169 double variant nucleosomal sequences (Figure 1); and clear periods of 2, 4, 6, 8 in the set of 348 H3.3-only nucleosomal sequences (Figure 2) while the set of 894 H2A.Z-only nucleosomal sequences does not exhibit this feature (Figure 3).

The enrichment (and possibly periodic patterns) of CA/TG dinucleotides in the genomes of many eukaryotic organisms can be resulted from CpG-methylation-deamination process, which dominates point substitutions in vertebrates [49], in which highly-methylated CpG dinucleotide is deaminated and then mutated to CA or TG [50,51]. This may lead to the possibility that the observed periodicities of CA/TG are caused by random deposition of H3.3-containing nucleosomes to CA/TG-enriched regions generated by this process. However, a recent work [52] has reported that H3.3 variant preferentially targets CpG-rich promoters and a large number of H3.3 promoters has low DNA methylation level. Taken together with this, our result suggests that the periodicities of CA/TG dinucleotides may play important roles in determining the target sites of nucleosomes containing H3.3 (both H3.3-only and double) variant. Also, from Table 2 we can see that the enrichment of CA/TG dinucleotides is one of the strongest discriminative signals distinguishing target sites of double variant nucleosomes from those of H3.3-only nucleosomes. No DNA motif, however, shows dominant effect on this target site selection. This suggests that the role of DNA sequence may be modest compared with that of other factors (such as chromatin remodeling complexes, PTMs) in identifying exact locations for double variant nucleosomes.

Previous work has shown that in both *D. melanogaster* and human cells, H3.3 is deposited into transcribed genes, promoters and gene regulatory elements [6]. It is incorporated into genes upon transcription induction and is associated with transcriptional elongation [24]. Recent *in vitro* study about the effects of histone variants on transcription reported, however, that the presence of H3.3 is transparent to the transcription and the effect of “hybrid” particles containing both H2A.Z and H3.3 (i.e. double variant nucleosomes) was entirely dominated by the presence of H2A.Z [53]. These results, together with what was reported in Jin et al. [32], have raised important questions that: why double variant nucleosomes are present at important regulatory regions and whether or not H3.3 simply marks sites of nucleosome destabilization or is itself important for mediating the process [54]. Our result here provides a complementary answer to these questions: the presence of H3.3 in double variant nucleosomes can help to position the particles at specific locations in promoters; based on that, H2A.Z, which is also included in the particles, can execute its functions on transcription process. This is consistent with the hypothesis that the process incorporating H3.3 into nucleosomes, which involves histone chaperones and nucleosome remodelers, also facilitates H2A.Z incorporation [54]; and with the *in vitro* result that H3.3 may be just a marker of chromatin regions in flux while the presence of H2A.Z directly affects the transcriptional properties of the particles [53].

## Conclusions

Histone variants, such as H2A.Z and H3.3, play significant biological roles in regulating chromatin structure and chromatin-based processes thereof. So it is important to understand how they are targeted to the deposited sites. Although it has been known that there are several biological pathways related to the deposition of these variants onto chromatin, the mechanisms are still unclear. Our work here shows that, there may exist a DNA-related mechanism regulating the deposition of different histone variants onto chromatin. In that, highly flexible dinucleotides, such as CA/TG, play remarkable role in the selection of deposited sites for H3.3-containing (both H3.3-only or double variant) nucleosomes. Moreover, recent works have also shown that H3.3-containing nucleosomes are deposited to sites marked with active PTMs (such as H3K4Me3) [55] and this deposition is controlled by different factors depending on specific genomic regions [52]. Future studies therefore should seek to understand how those factors coordinate to regulate the deposition of H3.3 variant onto chromatin and its relationship with transcription process.

## Methods

### Data preparation

#### Nucleosome positioning analysis

Experimental ChIP-Seq data (BED files) corresponding to “H2A.Z only”, “H3.3 only” and Double (H2A.Z/H3.3) histone variants from human H3.3 HeLa S3 cell were received from [32]. Nucleosome positioning profiles of those histone variants were identified using NPS [56] with default settings except that the parameter “minimum nucleosome length” was set to 100bp. We received totally 14565 double variant, 4876 H2A.Z-only and 2799 H3.3-only nucleosomes. The lengths of these nucleosomes were then truncated/extended, centered on the nucleosome, to 150bp if the resultant lengths were longer/shorter than 150bp. Genomic sequences corresponding to these nucleosomes were extracted from UCSC Genome Browser [57], human genome Build 36.1 (hg18 assembly). Repeats from RepeatMasker and Tandem Repeat Finder were excluded in our analysis.

#### The gene set

UCSC Old Known Genes were extracted and then mapped to Affymetrix U133P2 probe IDs using the tables provided in the UCSC Genome Browser [58]. Genes without corresponding U133P2 IDs were removed. If multiple genes map to the same U133P2 ID, only one was retained. We also removed genes from chromosomal regions marked with “random” or genes from haplotype regions. The final set contained 18285 genes.

#### Training datasets

All nucleosomes identified above were mapped to the vicinity of the transcription start sites (TSSs) (10000bp upstream and 2000bp downstream) of the genes in the gene set. Only nucleosomes belonging to these regions were used for further analysis. After this step, we received 2169 double variant, 894 H2A.Z-only and 348 H3.3-only nucleosomes. We then created two sequence datasets, namely *doulbe-h2az* and *double-h33,* for training purpose. *double-h2az* dataset contained 800 double variant nucleosomal sequences, selected randomly from 2169 double variant nucleosomes, and 800 H2A.Z-only nucleosomal sequences, selected randomly from 894 H2A.Z-only nucleosomes. Similarly, *double-h33* dataset contained 300 double variant nucleosomal sequences, selected randomly from 2169 double variant nucleosomes, and 300 H3.3-only nucleosomal sequences, selected randomly from 348 H3.3-only nucleosomes above.

### Support vector machine (SVM) classifiers

Given a training set containing instance-class pairs (x*_i_*, *y_i_*), *i* = 1, 2, ⋯, *l* where x*_i_* ∈ *R^l^* and *y_i_*  ∈ {–1, 1} is a class label, an SVM classifier is a hyperplane w*^T^ϕ*(x*_i_*) + *b*, where *ϕ*(x*_i_*) is a function mapping x*_i_* into a higher (maybe infinite) dimensional space, that best separates the two classes. The hyperplane is obtained by solving the following primal optimization problem:(1)

Its dual is a quadratic optimization problem:(2)

where e is an unit vector, *C >* 0 is an error penalty parameter, *Q_ij_ = y_i_y_j_K* (x*_i_*,x*_j_*), *K* (x*_i_*,x*_j_*) = *ϕ*(x*_i_*)*^T^ ϕ*(x*_j_*) is a kernel function. In our work, we employed *Radial Basis Function (RBF)* kernel *K* (x*_i_*,x*_j_*) = *exp*(–(x*_i_* – x*_i_*)^2^) to build SVM classifiers. Discriminant value for a testing instance x given by a trained classifier is: *f*(x) = *∑_i_ α_i_y_i_K*(x,x*_i_*) + *b.* Gist software package [59] was utilized for the tasks of training and testing SVM classifiers.

### Feature selection with Fisher criterion

Feature selection is a process of selecting a subset of relevant features available from the data that most contribute to distinguishing instances from different classes. In our method, sequence features related to two kinds of histone variants, double variant and H3.3 only or double variant and H2A.Z only, were identified and ranked by their Fisher scores (or F-score in short). This is one of statistical criteria that is simple, effective and independent of the choice of classification method. The discriminative strength of each feature is defined as following:

Given a dataset *X* with two classes, denote instances in class 1 as *X*^1^*,* and those in class 2 as *X*^2^*.* Assume  is the average of the *jth* feature in *X^k^,* the F-score of the *jth* feature is:(3)

where(4)

The numerator indicates the discrimination between two classes, and the denominator indicates the scatter within each class. The larger the F-score is, the more likely this feature is more discriminative. Gist software package [59] was used to calculate F-scores for different DNA motifs.

### Autocorrelation analysis

Correlation functions measure the enrichment of certain pairs of motifs at a distance of *k* bp. To calculate *XX*-autocorrelation fuction we followed the method described in [48]. Given a motif *X* and a DNA sequence *S,* we count in the entire *S* the number  of pairs of two identical motifs *X* and *X* separated by *k* base pairs. There are *L* – *k* – *l* + 1 pairs in a sequence of length *L*, where *l* is the length of the motif. Consequently, the probability to find the pair *X* – *X* at the distance *k* can be estimated as:(5)

The probability to find a single motif *X,* denoted by *P_X_*(*k*)*,* can be estimated as:(6)

where  is the number of motif *X* in the sequence *S.* If the pairs at a distance *k* are statistically independent we have: *P_XX_*(*k*) = *P_X_*(*k*) * *P_X_*(*k*). Thus the difference, *C_XX_*(*k*) = *P_XX_*(*k*) – *P_X_*(*k*) * *P_X_*(*k*), measures the correlation at a distance of *k* base pairs. A positive peak of the covariance *C_XX_*(*k*) implies that there are more *X* – *X* pairs at a distance of *k* than expected by chance. The mean covariance function for a set of sequences was calculated by averaging individual functions over all the sequences in the set.

## Competing interests

The authors declare that they have no competing interests.

## Authors’ contributions

NTL and TBH defined the research problem. NTL and BHH designed the experiment. NTL, BHH and TBH drafted the manuscript. All authors contributed to and approved the final version of the manuscript.
